# Assessing routes of hepatitis C transmission in HIV-infected men who have sex with men using single genome sequencing

**DOI:** 10.1371/journal.pone.0235237

**Published:** 2020-07-15

**Authors:** Hui Li, Kristen M. Marks, Andrew H. Talal, Wouter O. van Seggelen, Bisher Akil, Asa Radix, Shirish Huprikar, Andrea D. Branch, Shuyi Wang, George M. Shaw, Daniel S. Fierer

**Affiliations:** 1 Department of Medicine, Perelman School of Medicine at the University of Pennsylvania, Philadelphia, Pennsylvania, United States of America; 2 Division of Infectious Diseases, Department of Medicine, Weill Cornell Medical College, New York, New York, United States of America; 3 Division of Gastroenterology and Hepatology, Department of Medicine, Weill Cornell Medical College, New York, New York, United States of America; 4 Division of Infectious Diseases, Department of Medicine, Icahn School of Medicine at Mount Sinai, New York, New York, United States of America; 5 Chelsea Village Medical, New York, New York, United States of America; 6 Callen-Lorde Community Health Center, New York, New York, United States of America; 7 Division of Liver Diseases, Icahn School of Medicine at Mount Sinai, New York, New York, United States of America; University of Cincinnati College of Medicine, UNITED STATES

## Abstract

The epidemic of hepatitis C virus (HCV) infection among HIV-infected men who have sex with men (MSM) is in its second decade, but the routes of transmission remain poorly understood. We hypothesized that by pairing single genome sequencing (SGS), to enumerate infecting HCV genomes (viruses), with detailed sexual and drug histories, we could gain insight into the routes of transmission among MSM. We used SGS to analyze blood specimens from eight HIV-infected MSM who had 10 episodes of acute (seronegative) or early HCV infections. Seven of eight men reported condomless receptive anal intercourse (CRAI), six with rectal exposure to semen, and all eight denied rectal trauma or bleeding. Of the 10 HCV infections, eight resulted from transmission of a single virus; one infection resulted from transmission of either one or a few (three or four) closely-related viruses; and one infection resulted from transmission of >10 distinct viruses. The participant infected by >10 viruses reported sharing injection equipment for methamphetamine during sex. Two other participants also injected methamphetamine during sex but they did not share injection equipment and were infected by a single virus. Conclusions: Most HCV infections of HIV-infected MSM without a history of either rectal trauma or bleeding or shared injection equipment were caused by a single virus. Intra-rectal exposure to semen during CRAI is therefore likely sufficient for HCV transmission among MSM. Conversely, rectal trauma or bleeding or shared injection equipment are not necessary for HCV transmission among MSM. These results help clarify routes of HCV transmission among MSM and can therefore help guide the design of much-needed behavioral and other interventions to prevent HCV transmission among MSM.

## Introduction

Although the epidemic of hepatitis C virus (HCV) infection among HIV-infected men who have sex with men (MSM) is in its second decade [1], the routes of HCV transmission remain poorly understood and highly controversial [2], largely for two reasons. First, multiple questionnaire-based studies from different countries yielded discordant results [3–9]. For example, several studies internationally found that condomless receptive anal intercourse (CRAI) was a significant risk factor for HCV infection [3,4,6,7,9], however, a German study [5] found that CRAI was not a significant risk factor for HCV infection, but that frequent rectal bleeding was. In contrast, a Belgian study [8] found that neither rectal bleeding nor CRAI were significant risk factors for HCV infection, but that douching prior to anal intercourse was, while a Dutch study [9] found that neither douching nor rectal bleeding were significant risk factors for HCV infection.

Second, HCV has long been considered an exclusively blood-borne pathogen and therefore that blood exposure is required for HCV transmission. But, as described above, the available evidence does not support that blood exposure is required for sexual transmission of HCV. Further, as neither penises nor fists typically bleed during sex, there is no plausible source of blood to cause transrectal HCV infection. But, as is the case with HIV, HCV is present in semen [10,11] and rectal fluid [12, 13], which are deposited in the rectum of MSM during sex. Unlike sexual transmission of HIV, however, which is mediated by receptors on various cells within the anogenital tract, transmission of HCV is mediated by binding to receptors on the surface of hepatocytes [14]. For sexual transmission of HCV to occur, HCV deposited in the rectum would need to cross a disrupted rectal mucosal barrier to enter the portal and/or systemic venous system. Therefore, to better understand the mechanisms of sexual transmission of HCV it is necessary to functionally characterize the rectal mucosal disruption during sex.

Single genome sequencing (SGS), which assesses the restriction in the genetic diversity of an infecting virus that is imposed by the mucosal (or cutaneous) barrier (“bottleneck”), has been a powerful tool to investigate routes of sexual transmission of HIV [15–19]. Sexual transmission of HIV among both women and MSM is mediated by low-multiplicity fluids such as semen, and in the case of MSM, also rectal fluid. Using SGS, we previously demonstrated that the number of infecting viruses (also referred to as transmitted/founder genomes) is correlated with the intactness of mucosal barriers exposed to these fluids among both women and MSM. Most women acquire a single virus during vaginal intercourse [16–18], but women with inflammation and decreased integrity of the vaginal mucosal barrier are more likely to acquire multiple viruses [17]. MSM, while usually infected by a single virus, are more likely to be infected by multiple viruses than are women [19]. This difference is likely due, at least in part, to the relatively larger disruptive effect of the inserted penis on the rectal mucosal barrier compared to the vaginal mucosal barrier. In contrast to HIV transmission due to low multiplicity sexual body fluids, people who inject drugs (PWID) are routinely exposed to blood, a high multiplicity source, that is contained in shared injection drug equipment. Consequently, most PWID are usually infected by more than one virus [20]. Taken together, these SGS studies investigating HIV transmission suggest that the number of infecting viruses can be used both as a surrogate for the intactness (or disruption) of the mucosal barrier onto which semen or rectal fluid containing HIV is deposited, as well as to distinguish between sexual and parenteral routes of infection.

To investigate similar questions about HCV infection, we adapted our SGS techniques to analyze a cohort of anonymous plasma donors with incident HCV infection and found a wide range (one to > 37) of infecting viruses [21]. We could not directly assess the association between the number of infecting viruses and routes of acquisition in these anonymous donors, however, although surreptitious injection drug use is common in this population [22]. We have now extended this evaluation to the epidemic of HCV infection among HIV-infected MSM. We utilized our well-characterized cohort of HIV-infected MSM with incident HCV infection about whom we have detailed HCV risk factor data including rectal exposure to semen and routes of sexualized drug use [4]. We hypothesized that men who had CRAI without rectal trauma or bleeding would have a relatively intact rectal mucosal barrier and would therefore be infected with a single/few viruses. Conversely, those who shared injection equipment during sexualized drug use would be infected with a high number of viruses.

## Materials and methods

### Study participants

HIV-infected MSM referred to the Mount Sinai and Weill Cornell Medical Centers for the management of newly-acquired HCV infections were enrolled in this study. Written informed consent was obtained with approval of the Institutional Review Boards of the Mount Sinai School of Medicine (subsequently “Icahn School of Medicine at Mount Sinai”) and the Weill Cornell Medical College in accordance with the Helsinki Declaration of 1975, as revised in 2000.

Upon enrollment, detailed interviews about sexual and drug-use practices were performed for each participant [4] by either Dr. Fierer at Mount Sinai or Dr. Marks at Cornell. We defined the concurrence of sex and methamphetamine use as sexualized drug use, as previously described [4], and we further distinguished between non-injection and injection drug use. Serial blood specimens were collected beginning at enrollment; serum and/or plasma was stored at -80°C until analyzed. In those treated for HCV during the study period of 2008 to 2012, treatment was with pegylated interferon plus ribavirin, the available treatment at the time of these studies, as described previously [23].

We defined the early stages of primary HCV infection based on antibody (Ab) serological response, generally in parallel with the definition of the early stages of HIV infection [24]. We therefore defined acute HCV as the period between HCV infection and Ab seroconversion, and defined early HCV as the limited period beginning at seroconversion, during which time robust ALT elevation (i.e. > 5x upper limit of normal) and/or wide fluctuation in HCV RNA (i.e. > 1 log_10_ IU/mL) occurs [1]. We defined the clinical onset of HCV infection, both in the case of primary infection and of superinfection, as the date of detection of either new HCV viremia or new >5-fold ALT elevation. We defined superinfection as detection of a new HCV strain that emerged prior to cure of the primary infection, as confirmed by SGS.

We used the 3^rd^ generation HCV enzyme immunoassay version 2.0 (Abbott Laboratories) to assess HCV serologic status; and the COBAS Ampli-Prep/COBAS TaqMan CAP/CTM [Roche Diagnostics; lower limit of quantification (LLOQ) 43 IU/mL] to quantify HCV RNA. We used the COBAS AmpliPrep/COBAS TaqMan HIV-1 Test, version 2.0 (Roche Diagnostics; LLOQ 48 copies/mL and subsequently 20 copies/mL) to quantify HIV RNA.

### Single genome sequencing

We amplified 5’ half genomes of HCV from serum or plasma samples from each participant by nested polymerase chain reaction (PCR) and performed SGS as previously described [15,21]. Due to the significant sequence heterogeneity among HCV genotypes, we used primer sets specific to the HCV genotype being analyzed, as follows:

Genotype 1a primer set: 5’ half genotype 1a: 1st round sense primer 1.core.F1 5’- ATGAGCACGAATCCTAAACCTCAAAGA- 3’, and 1^st^ round antisense primer 1.NS4A.R1 5’-GCACTCTTCCATCTCATCGAACTC-3’; 2nd round sense primer 1.core.F2 5’-TCAAAGAAAAACCAAACGTAACACCAACCG-3’, and 2nd round antisense primer 1.NS3A A.R2 5’- AGGTGCTCGTGACGACCTCCAGG-3’.

Genotype 1b primer set: 5’ half genotype 1b: 1st round sense primer 1bCORE-F1 5’- ATGAGCACGAATCCTAAACCTCAAAGA- 3’, and 1^st^ round antisense primer 1bNS4A-1 5’-GCACTCTTCCATCTCATCGAACTC-3’; 2nd round sense primer 1bCORE-F2 5’-TCAAAGAAAAACCAAACGTAACACCAACCG -3’, and 2nd round antisense primer 1bNS3-R2 5’-GGTGCTCGTGACGACCTCCAGGTC -3’.

Genotype 2b primer set: 5’ half genotype 2b: 1st round sense primer 2bCORE_F1 5’- ATGAGCACAAATCCTAAACCTCAAAGA-3’, 1^st^ round antisense primer 2BNS4AR1 5’-TTCTTCCATCTCATCAAAGGCCTCA-3’; 2nd round sense primer 2bCORE-F2 5’- AATCCTAAACCTCAAAGAAAAACCAAAA-3’, and 2nd round sense primer 2BNS4AR2 5’-CCAGGACCCATGTGCTTGTCAT-3’.

The phylogenetic analysis was performed as previously described [21]. We first aligned the 5’ half-genome sequences with Geneious Prime software and then hand-checked them to improve the alignments according to the codon translation. The sequences were analyzed using a sequence visualization tool, Highlighter (www.HIV.lanl.gov) [16], that allows tracing of common ancestry between sequences based on individual nucleotide polymorphisms. The nucleotide diversity of HCV in each participant was analyzed by maximum-likelihood phylogenetic analyses using the PhyML program (www.atgc-montpellier.fr/phyml) [25]. To identify and enumerate candidate infecting viruses, we used Poisson fitter tools as well as two mathematical models that we developed previously to analyze the Hamming distance within each participant and to analyze HCV sequence diversity [21,26].

### Mathematical models

The significant diversification that occurs during HCV replication (“quasispecies”) is due to two RNA-dependent RNA polymerase (RdRp) copying events. HCV RdRp is a virally encoded error-prone polymerase with an estimated mutation rate of about 2.5 x 10^−5^ per base per generation [27]. The original positive strand along with newly created negative strands plus newly expressed non-structural proteins form a cytosolic replication complex (RC) that serves as a template for producing new positive strand RNA. Based on these replication mechanisms, the first mathematical model we developed predicts that the early HCV diversification is random during acute infection; pairwise sequence differences (Hamming distance) follow a Poisson distribution; and sequence changes exhibit star-like phylogeny with the coalescence corresponding to a single infecting virus. Our previous studies corroborated this model, demonstrating that HCV diversification is essentially random during the early linear growth phase [21,26,27]. The pattern may change, however, after the early linear growth phase, when the RC accumulate over many generations and produce viruses from the different RNA templates in long-lived hepatocytes. These descendants are produced from different generations of RC, and small numbers of shared mutations can arise prior to the onset of immune selection [27]. The second mathematical model we developed addresses these replication mechanisms and predicts the emergence of shared stochastic mutations as virus growth plateaus, which can lead to occasional violations of a Poisson distribution or star-like phylogeny of mutations. In our conservative modeling, we defined a cluster of sequences with >4 shared mutations in a half-genome (~5,000 bp) as unambiguously to have arisen from a different infecting variant [21]. This alternative model prediction allows us to distinguish closely related infecting HCV lineages from sequences that evolve from a single infecting virus but that share early stochastic mutations. We used these mathematical models and Poisson fitter tools to enumerate infecting viruses and analyze the Hamming distance within each participant [21,26].

## Results

### Study participants

We enrolled eight HIV-infected MSM with acute or early primary HCV infection between October 2008 and March 2012. Their age range at the time of HCV diagnosis was 29 to 47 years; six were white, one was Hispanic, and one was black. Their CD4 counts ranged from 130 to 911 cells/μL. Four were not taking antiretroviral treatment due to the current treatment recommendations [28], personal choice, and/or complications of addiction (Table 1).

**Table 1:** Demographic and behavioral characteristics of eight HIV-infected men who have sex with men with acute HCV infection.

Participant	Age (years)	Race	Duration known HIV infection (years)	CD4 count (cells/μL)	Taking ART	HIV RNA (log_10_ IU/mL)	Sexual risk factors	Sexualized drug use	Reason for HCV testing	Clinical onset criterion
1	42	H	8	437	no	3.0	CRAI with semen in rectum	meth, inject, no share (1° infect); none (super-infect)	elevated ALT	ALT 394 U/L
2	35	W	7	445	no	4.2	CRAI with semen in rectum	meth, no inject	elevated ALT	ALT 299 U/L
3	29	B	9	210	yes	2.4	CRAI with semen in rectum	meth, no inject (1° infect); none (super-infect)	CRAI	HCV RNA 7.4 log_10_ IU/mL
4	27	W	3	630	yes	<1.7	CRAI; unknown if semen in rectum	none	elevated ALT	ALT 330 U/L
5	30	W	3	509	no	4.8	CRAI with semen in rectum	meth, inject, no share	meth, inject	HCV RNA 7.5 log_10_ IU/mL
6	47	W	6	433	yes	<1.3	insertive anal intercourse	none	elevated ALT	ALT 984 U/L
7	37	W	8	130	no	5.5	CRAI with semen in rectum	meth, inject, known share	meth, inject	HCV RNA 6.6 log_10_ IU/mL
8	40	W	10	911	yes	1.8	CRAI with semen in rectum	none	elevated ALT	ALT 537 U/L

ART, antiretroviral therapy; RNA, ribonucleic acid; HCV, hepatitis C virus; H, Hispanic; W, white; B, black; ALT alanine aminotransferase; U/L, units per liter; IU, international units per liter; mL, milliliter; meth, inject, no share, injection of methamphetamine without sharing of injection equipment; 1° infect, primary infection; CRAI, condomless receptive anal intercourse; super-infect, super-infection

**Fig 1:**
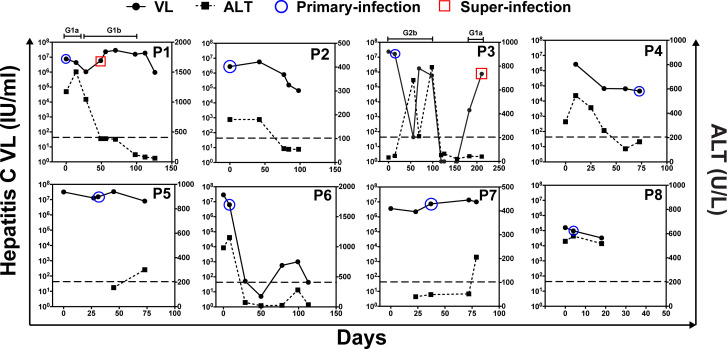
Evolution of ALT and hepatitis C virus (HCV) RNA levels and samples selected for single genome sequencing (SGS) after clinical onset of acute HCV infection among eight HIV-infected men who have sex with men. HCV RNA levels (log_10_ IU/mL) (dots) are plotted against the left y-axis; ALT levels (U/L) (filled squares) are plotted against the right y-axis; and time (weeks) from clinical onset of HCV infection is plotted on the x-axis. All available measurements during the sampling period are shown. The samples analyzed using SGS from the primary HCV infections are shown by a blue circle around the corresponding HCV VL point. The samples analyzed using SGS from the HCV superinfections (P1 and P3 only) are shown by a red square around the corresponding HCV VL point. The bars above the plots for P1 and P3 show the duration of the viremia from the primary and superinfections, labeled by their genotype. SGS, single genome sequencing; P, participant; RNA, ribonucleic acid; IU, international units; mL, milliliter; ALT, alanine aminotransferase; G1a, genotype 1a; G1b, genotype 1b; G2b, genotype 2b.

**Fig 2:**
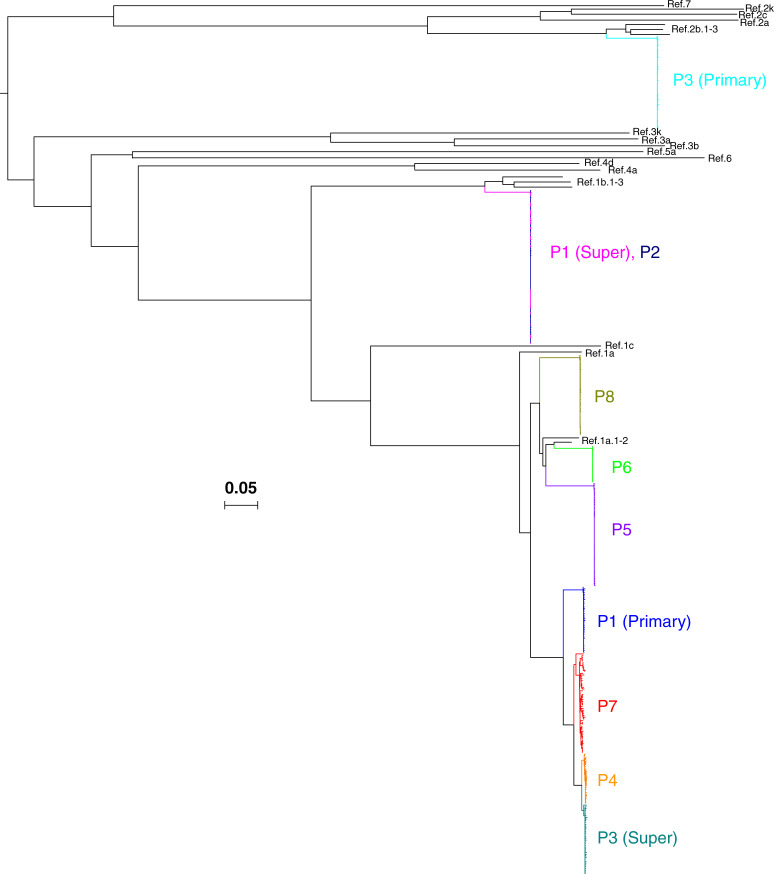
Maximum-likelihood tree of HCV sequences during acute and early hepatitis C virus (HCV) among eight HIV-infected men who have sex with men. 5’ half genome sequences corresponding to core, E1E2, p7, NS2, and NS3 genes derived from 10 HCV infections from eight participants are shown along with HCV genotype 1 to 7 reference sequences (black). Two participants (P1 and P3) had both primary and superinfections. P1 (super) and P2 are an epidemiologically linked transmission pair. The sequences from each infection was highlighted with different color. The horizontal scale bar indicates 5% diversity. P, participant; Ref., reference.

The diagnosis of primary HCV infection was suggested by new ALT elevation during routine HIV care in five of the eight men, and by HCV RNA screening in the other three men due to behaviors considered high risk for HCV acquisition. All men had anal intercourse in the three months prior to HCV acquisition; seven had CRAI, six of whom had intra-rectal exposure to semen. Some men continued to have CRAI with intra-rectal exposure to semen during the post-infection period, including during interferon treatment for their primary HCV infection, and two of the eight men became superinfected before the initial infection cleared. One (P1) was diagnosed with superinfection in retrospect, through sequencing of serial specimens (Figs 1 and 2). The other (P3) had a virologic breakthrough during interferon treatment of his primary HCV infection that was determined to be a superinfection by sequencing (Figs 1 and 2). We therefore evaluated a total of 10 HCV infections in eight men in this study.

At the time of primary HCV infection, the HCV Ab test result at clinical onset was negative in six of the eight men; positive in one; and one participant (P4) had a negative Ab test five weeks prior to clinical onset and a repeat Ab test was not obtained. Among the six men who had a negative Ab at clinical onset, at the time the blood sample used for SGS was collected, three still had a negative Ab test; one had an equivocal Ab test; and two had a newly positive Ab test, having had a negative Ab test one to five weeks earlier. The median time between clinical onset and the blood sample used for SGS was four (range 0 to 10) weeks (Table 2, Fig 1).

**Table 2:** Single genome sequencing (SGS) results for 10 acute hepatitis C virus infections of eight HIV-infected men who have sex with men.

Participant	HCV Ab at clinical onset	Clinical onset to SGS (weeks)	HCV Ab at SGS	HCV RNA at SGS (log_10_ IU/mL)	HCV genotype	5' half-genome sequences (No)	Nucleotides in 5' half sequences (No)	Diversity (%)		Poisson goodness of p-value	Star-like phylogeny	infecting viruses (No)	Transmission risk(s)
								Mean (range)	Median				
1 primary	negative	3	positive	6.9	1a	37	4905	0.16 (0.04–0.33)	0.16	0.719	no	1	CRAI+semen; inject meth-share
1 super-infection	positive	0	positive	6.8	1b	56	5059	0.05 (0.00–0.12)	0.04	0.375	yes	1	CRAI+semen
2	negative	2	negative	6.4	1b	30	4905	0.06 (0.00–0.16)	0.06	0.664	yes	1	CRAI+semen
3 primary	negative	2	equivocal	7.2	2b	53	4940	0.10 (0.00–0.32)	0.10	0.002	yes	1	CRAI+semen
3 super-infection	positive	4	positive	5.9	1a	39	4905	0.22 (0.10–0.69)	0.20	2 x 10^−16^	yes	1	CRAI+semen
4	negative 4 weeks prior	10	not done	4.7	1a	28	4917	0.24 (0.00–0.41)	0.24	2 x 10^−16^	no	1, or 3 or 4 closely related	CRAI unkn semen
5	negative	5	negative	7.2	1a	58	4904	0.06 (0.00–0.18)	0.06	0.248	yes	1	CRAI+semen; inject meth-share
6	positive	1	positive	6.8	1a	20	4904	0.05 (0.00–0.12)	0.04	0.241	yes	1	insertive anal intercourse
7	negative	5	negative	6.9	1a	56	4904	0.62 (0.00–1.79)	0.61	NA	NA	>10	CRAI+semen; inject meth+share
8	negative	5	positive	5.0	1a	45	4902	0.09 (0.00–0.18)	0.08	0.034	no	1	CRAI+semen

HCV, hepatitis C virus; Ab, antibody; RNA, ribonucleic acid; IU, international units; mL, milliliter; SGS, single genome sequencing; CRAI+semen, condomless receptive anal intercourse with semen received into the rectum; CRAI unkn semen, condomless receptive anal intercourse but unknown if semen received into the rectum; meth, methamphetamine; NA, not applicable; inject meth-share, injection of methamphetamine without sharing of injection equipment; inject meth+share, injection of methamphetamine with sharing of injection equipment

### SGS analysis

The HCV RNA levels of the samples analyzed by SGS ranged from 4.7 to 7.2 log_10_ IU/mL. A total of 422 single genome sequences corresponding to core, E1E2, p7, NS2, and NS3 genes derived from 10 HCV infections were subjected to maximum-likelihood phylogenetic analysis (GenBank accession numbers: MT416764 to MT417181). Sequences formed participant-specific clades and clustered with reference sequences of genotypes 1a (n = 7), 1b (n = 2), and 2b (n = 1) (Fig 2). Sequences from participants 1 and 2 had previously been shown to be epidemiologically linked [26].

**Fig 3:**
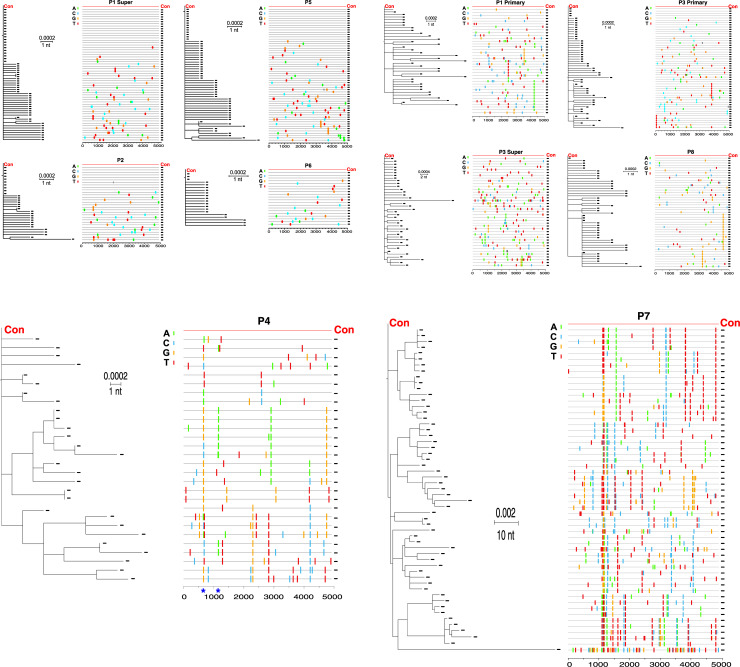
Four patterns of sequence diversity among 10 acute and early hepatitis C virus (HCV) infections in eight HIV-infected men who have sex with men. Maximum-likelihood trees (left) and Highlighter plots of 5’ half genome (core, E1E2, p7, NS2, and NS3) (right) are shown as paired for each infection. For each infection, the consensus sequence (red) was used as the root and the master template in the ML tree and the Highlighter plot, respectively. The sequences in the Highlighter plots are displayed in the same order as the taxa in the Maximum-likelihood trees. A) P1 superinfection, P2, P5, and P6 showed very low sequence diversity and star-like phylogeny, consistent with acquisition of a single founder virus; B) P1 primary infection, P3 primary and superinfections, and P8. These showed very low sequence diversity but had minor deviations from either a Poisson distribution or star-like diversity, but consistent with acquisition of a single founder virus; C) P4 showed very low sequence diversity but had many shared nodes in the phylogenetic tree. The blue stars at nucleotide position 700–708 and 1198–1203 (H77) indicate the amino acid changes at glycosylation site 234 in E1 and residues 400/401 in HVR1 of E2, sites of strong selection. Both nucleotide and amino acid positions were named based on H77 reference sequence. These patterns were most consistent with acquisition of one, or a few (three or four) closely related viruses; and D) P7 showed very high diversity and demonstrated a very complex sequence evolution, most consistent with acquisition of >10 infecting viruses. P, participant; nt, nucleotide; E1, envelope glycoprotein 1; HVR1, hypervariable region 1; A, adenine; C, cytosine; T, thymine; G, guanine.

We found four distinct patterns among the viral sequences from the 10 infections. The first pattern, found in four infections (P1 superinfection, P2, P5, and P6), was exemplified by the superinfection of P1, with extremely homogeneous sequences of very low diversity (mean = 0.05%; minimum = 0.00%; maximum = 0.12%) (Table 2). In addition, all sequences from the superinfection of P1 formed a single monophyletic discrete low-diversity lineage in the phylogenetic tree, which exhibited typical star-like phylogeny (not shown), and all the mutations appeared randomly in their corresponding Highlighter plots (Fig 3A). Among the 56 sequences, 36% were identical and 25%, 20%, and 12.5% contained 1, 2, and 3 nucleotide changes, respectively, and coalesced into a single unambiguous consensus sequence representing the infecting virus sequence. The patterns found in the infections of P2, P5, and P6 were overall similar, including the maximum intra-lineage diversities, which ranged from 0.12% to 0.18% (Table 2, Fig 3A). Based on these multiple criteria, we therefore concluded that these four infections in four participants (P1 superinfection, P2, P5, and P6) were due to a single infecting virus. All four of these men denied sharing of injection equipment but participated in CRAI; three had intra-rectal exposure to semen without rectal trauma or bleeding, one of whom (P5) additionally reported injecting methamphetamine during sexualized drug use (but denied sharing of injection equipment) (Table 1).

The second pattern was found in four infections in three men (P1 primary infection, P3 primary and superinfections, and P8). Similar to the sequences exhibiting the first pattern, these second-pattern sequences had very low diversity (mean: 0.09% to 0.22%; minimum: 0.00% to 0.10%; maximum: 0.18% to 0.69%) (Table 2), but they deviated from either a Poisson distribution or star-like phylogeny or both due to a small number of shared mutations (Fig 3B). Using our conserved model, which estimates that fewer than four observed shared mutations can arise from a single infecting virus, we concluded that a single infecting virus likely resulted in these four sexual transmissions, including one (P1 primary infection) who injected methamphetamine but did not share injection equipment (Table 1).

The third pattern was seen only in P4. The sequences also exhibited very low diversity (mean: 0.24%; minimum: 0.00%; maximum: 0.41%) (Table 2) but with many closely related subsets that shared multiple polymorphisms that appeared as shared nodes in the phylogenetic tree and multiple stripes in the Highlighter plot (Fig 3C). Strong selection was observed at glycosylation site 234 (H77) in E1 and residues of 400/401 (H77) in HVR1 of E2, which are reported to be autologous neutralizing antibody targeting sites [29–31] (Fig 3C; the two sites are shown as stars at the base of the Highlighter plot). This pattern can be explained in two ways: 1) a single infecting virus with extensive random diversification and accumulation of shared mutations, and emergence of neutralizing antibodies leading to strong amino acid selection in E1E2 region over the relatively long period (10 weeks) between the clinical onset of infection and the time when the SGS specimen was obtained; or 2) acquisition of three to four closely related viruses from an individual who himself was recently infected by a single virus or by closely related viruses. This man had CRAI without rectal trauma or bleeding, and without injection of methamphetamine (Table 1).

Finally, in contrast to the low diversity of infecting viruses identified among the other nine infections of seven participants, the fourth pattern, seen only in sequences from P7, showed very high diversity (mean: 0.62%; minimum: 0.00; maximum: 1.79%) (Table 2), non-homogeneity, and exceeded the diversity cut-off for Poisson Fitter and star-like phylogeny analyses. The phylogenetic tree and Highlighter plot analyses show the very complex sequence evolution (Fig 3D). These patterns are most consistent with acquisition of >10 infecting viruses by this man who, in addition to intra-rectal exposure to semen during CRAI, also reported sharing of injection equipment for methamphetamine as part of his sexualized drug use (Table 1).

In summary, we determined that at least eight of nine HCV infections were acquired in association with intra-rectal exposure to semen during CRAI, but without rectal trauma or bleeding and without sharing of injection equipment. These infections were the consequence of transmission and productive infection by a single virus, or possibly in one case, a few (three or four) viruses. The one man who reported only insertive anal intercourse may have been infected by HCV in rectal fluid. Of the three men who reported injecting methamphetamine as part of their sexualized drug use, only the one (P7) who reported actual sharing of injection equipment was infected by many (>10) viruses, while the other two (P1 primary, P5), who denied sharing injection equipment, were infected by a single virus.

## Discussion

To address the relatively poor understanding of and resultant controversies about the routes of HCV transmission among MSM, we built upon earlier studies that used SGS to study routes of HIV transmission to study these HCV transmissions. In this study, most HCV transmissions among HIV-infected MSM who had intra-rectal exposure to semen during CRAI were due to a single virus. These results suggest that subclinical abrasion by an inserted penis during CRAI is sufficient to allow HCV from semen to enter the rectal venous system, resulting in infection of the liver by a single virus. This is the manner in which MSM are presumed to be infected by hepatitis B virus, which is also present in semen [32] and also does not have receptors in the anogenital tract [33].

While our study demonstrates that rectal trauma or bleeding are not necessary for HCV transmission, more vigorous abrasion, and at the extreme, trauma resulting in frank rectal bleeding, would likely increase HCV transmission by further increasing access to the bloodstream of HCV deposited in the rectum. A Swiss study reporting that HIV-infected MSM inferred to have been infected by more than a single HIV founder virus, suggesting more rectal mucosal disruption, were more likely to subsequently acquire HCV [34] is supportive of this hypothesis. HIV infection itself could also potentially increase susceptibility to transrectal HCV infection due to the massive, persistent memory CD4 cell depletion from the colonic mucosa [35]. Colonic CD4 cell depletion results in a significantly more porous barrier to small molecules [36], which could include HCV contained in semen, or rectal fluid coating a penis, fist, or sex toy (i.e. fomite) [12]. The recent demonstrations of sexual HCV transmission among MSM taking pre-exposure prophylaxis against HIV [37–40] show that HIV infection is not a necessary predisposition for transrectal HCV infection, however.

Our study also suggests that the long-standing view that HCV is an exclusively blood-borne pathogen has not only pathologized sex among MSM who acquire HCV as “traumatic,” but has resulted in the conflation of the substantial HCV transmission risks of addictive drug use by PWID with the much lower HCV transmission risk of sexualized drug use by MSM. Sexualized drug use (including methamphetamine) among MSM occurs mostly on weekends and is typically done just once during the occasion, in association with sex [41]. In contrast, addictive use of heroin, cocaine, and (non-sexualized) methamphetamine, which frequently results in multiple daily exposures in PWID, is uncommon among MSM. In our experience, MSM who use methamphetamine routinely purchase their own injection equipment in pharmacies and have devised additional practices to prevent sharing their injection equipment (Fierer DS, unpublished results). In addition, injecting methamphetamine is simpler than injecting heroin or cocaine; methamphetamine is crystalized and highly water soluble [42], so no “cooking” or filtering paraphernalia are needed, decreasing the chances for blood contamination of injection equipment.

The limitations of our study should be considered. Our sample size and perhaps the local culture of sex practices in NYC limited our ability to assess more of the gamut of sexual exposures, such as group sex, shared toys, fisting, or overt rectal trauma or bleeding during sex. Larger studies could be done to investigate the relationship between these activities with different degrees of disruption of the rectal mucosal barrier and the number of founder viruses. Lack of detection of additional infecting viruses beyond the single virus found in most infections may have occurred, although we had a 90% power to detect variants present in 10% of the population [16]. Further, our finding of predominately a single infecting virus is supported by the three other small studies of sexually transmitted HCV infection in HIV-infected MSM in which infecting viruses were enumerated [43–45]. Also consistent with these studies [43,44], we found that one man may have acquired a few closely related viruses, which is consistent with “acute to acute” transmission, that is, transmission from a donor who was himself acutely infected. It has been hypothesized that HCV may be more transmissable in the pre-seroconversion acute phase, thereby favoring transmission by more than a single virus [10,11]. Rectal fluid could also be the HCV transmission source by directly infecting an inserted penis, or through fomite (e.g. sex toys) and fomite-like (e.g. fists and penises) mucosal exposures [12]. Only one of the nine men in our study reported having insertive anal intercourse as his sole risk factor (P6), so we could not adequately assess the role of exposures to rectal fluid. Our ability to clearly distinguish sexual from parenteral routes of infection was also limited by the small number of participants who injected methamphetamine. Our findings are, however, generally supported by two other studies in which likely parenteral exposures were associated with a larger number of transmitted viruses [21,46], and by a recent study showing that innumerable viruses were transmitted by the extremely high multiplicity exposures mediated by kidney or heart transplantations [47].

In summary, we found that most HCV infections in HIV-infected MSM without a history of either rectal trauma or bleeding or shared injection equipment were initiated from a single founder virus. Intra-rectal exposure to semen during condomless anal intercourse is therefore likely sufficient for HCV transmission among MSM. Conversely, rectal trauma or bleeding or shared injection equipment are not necessary for HCV transmission among MSM. These results help clarify routes of HCV transmission among MSM and can therefore help guide the design of much-needed behavioral and other interventions to prevent HCV transmission among MSM.
